# Bronchiectasis - Exercise as Therapy (BREATH): rationale and study protocol for a multi-center randomized controlled trial

**DOI:** 10.1186/s13063-022-06256-2

**Published:** 2022-04-11

**Authors:** Taryn Jones, Kerry-Ann F. O’Grady, Vikas Goyal, Ian B. Masters, Gabrielle McCallum, Christopher Drovandi, Thomas Lung, Emmah Baque, Denise S. K. Brookes, Caroline O. Terranova, Anne B. Chang, Stewart G. Trost

**Affiliations:** 1 Queensland University of Technology, Brisbane, QLD Australia; 2 Queensland Children’s Hospital, Brisbane, QLD Australia; 3 Gold Coast University Hospital, Gold Coast, QLD Australia; 4 The University of Queensland, Brisbane, QLD Australia; 5 Menzies School of Health Research, Darwin, NT Australia; 6 George Institute for Global Health, Sydney, NSW Australia; 7 Griffith University, Brisbane, QLD Australia

**Keywords:** Bronchiectasis, Pediatric, Physical activity, Exercise, Exacerbation, Randomized Controlled trial

## Abstract

**Background:**

Globally, bronchiectasis (BE) unrelated to cystic fibrosis (CF) is recognized as a major cause of respiratory morbidity, mortality, and healthcare utilization. Children with BE regularly experience exacerbations of their condition resulting in frequent hospitalizations and decreased health-related quality of life (HR-QoL). Guidelines for the treatment and management of BE call for regular exercise as a means of improving aerobic fitness and HR-QoL. Moreover, research in adults with BE has shown that exercise can reduce the frequency of exacerbations, a potent predictor of future lung function decline and respiratory morbidity. Yet, to date, the health benefits resulting from therapeutic exercise have not been investigated in children with BE. The BREATH, Bronchiectasis - Exercise as Therapy, trial will test the efficacy of a novel 8-week, play-based therapeutic exercise program to reduce the frequency of acute exacerbations over 12 months in children with BE (aged ≥ 4 and < 13 years). Secondary aims are to determine the cost-effectiveness of the intervention and assess the program’s impact on aerobic fitness, fundamental movement skill (FMS) proficiency, habitual physical activity, HR-QoL, and lung function.

**Methods:**

This multi-center, observer-blinded, parallel-group (1:1 allocation), randomized controlled trial (RCT) will be conducted at three sites. One hundred and seventy-four children ≥ 4 and < 13 years of age with BE will be randomized to a developmentally appropriate, play-based therapeutic exercise program (eight, 60-min weekly sessions, supplemented by a home-based program) or usual care. After completing the baseline assessments, the number of exacerbations and secondary outcomes will be assessed immediately post-intervention, after 6 months of follow-up, and after 12 months of follow-up. Monthly, parental contact and medical review will document acute respiratory exacerbations and parameters for cost-effectiveness outcomes.

**Discussion:**

The BREATH trial is the first fully powered RCT to test the effects of a therapeutic exercise on exacerbation frequency, fitness, movement competence, and HR-QoL in children with bronchiectasis. By implementing a developmentally appropriate, play-based exercise program tailored to the individual needs of children with bronchiectasis, the results have the potential for a major paradigm shift in the way in which therapeutic exercise is prescribed and implemented in children with chronic respiratory conditions. The exercise program can be readily translated. It does not require expensive equipment and can be delivered in a variety of settings, including the participant’s home. The program has strong potential for translation to other pediatric patient groups with similar needs for exercise therapy, including those with obesity, childhood cancers, and neurological conditions such as cerebral palsy.

**Trial registration:**

Australian and New Zealand Clinical Trials Register (ANZCTR) ACTRN12619001008112

## Administrative information

Note: The numbers in curly brackets in this protocol refer to SPIRIT checklist item numbers. The order of the items has been modified to group similar items (see http://www.equator-network.org/reporting-guidelines/spirit-2727-statement-defining-standard-protocol-items-for clinical-trials/).

**Table:** 

Title {1}	Bronchiectasis - Exercise as Therapy (BREATH): rationale and study protocol for a multi-center randomized controlled trial
Trial registration {2a and 2b}	Australian and New Zealand Clinical Trials Register (ANZCTR) number ACTRN12619001008112. The ANZCTR is recognized by the World Health Organization International Clinical Trials Registry Platform as a Primary Registry.
Protocol version {3}	10 August 2021, Version 4
Funding {4}	National Health and Medical Research Council Grant
Names, affiliations, and roles of protocol contributors {5a}	^1^Queensland University of Technology, Brisbane, QLD, Australia. ^2^Queensland Children’s Hospital, Brisbane, QLD, Australia. ^3^Gold Coast University Hospital, Gold Coast, QLD, Australia. ^4^The University of Queensland, Brisbane, QLD, Australia. ^5^Menzies School of Health Research, Darwin, NT, Australia. ^6^George Institute for Global Health, Sydney, NSW, Australia. ^7^Griffith University, QLD, Australia.
Name and contact information for the trial sponsor {5b}	Professor Stewart Trost Queensland University of Technology Centre for Children’s Health Research (CCHR) Level 4, 62 Graham Street South Brisbane QLD 4101 s.trost@qut.edu.au + 61 7 3069 7301
Role of sponsor {5c}	The study sponsor is responsible for the study design; collection, management, analysis, and interpretation of the data; writing of the report; and decision to submit the report for publication. The study sponsor will be responsible for the overall coordination of the trial and will work closely with the clinical chief/associate investigators to monitor the project staff and trial conduct at each site.

## Introduction

### Background and rationale {6a}

Bronchiectasis (BE) unrelated to cystic fibrosis (CF) is a major cause of respiratory morbidity and a significant contributor to health care utilization in children and adolescents globally [1–3]. It is the end point of the chronic suppurative lung disease (CSLD) continuum and is described as abnormal irreversible dilatation of the airways and associated with airway infection and inflammation [4, 5]. Children with BE regularly experience exacerbations of their condition (increased wetness and severity of cough, breathlessness, chest pain, and/or wheeze) resulting in frequent hospitalizations and decreased health-related quality of life (HR-QoL) [2, 6]. Prevalence data in children are scarce; however, a recent review of the epidemiology of CLSD estimated the prevalence of BE to range from 0.2 to 15 cases per 100,000 [7]. BE is particularly prevalent among socially disadvantaged populations, such as indigenous communities of Australia, New Zealand, Alaska, and Canada [7, 8]. A study of Central Australian Aboriginal children reported the prevalence as high as one in every 68 children [9].

The treatment goals of multi-disciplinary management are to prevent airway damage and optimize quality of life and minimize exacerbations [4]. Current clinical treatment guidelines recommend general exercise to improve aerobic fitness and HR-QoL [9]. However, the health impacts associated with therapeutic exercise in children with BE are not well understood [9, 10]. A recent meta-analysis of the effects of exercise training on physical and psychosocial health in children with chronic respiratory disease identified no published studies investigating the impact of therapeutic exercise in this population [11]. Therapeutic exercise may benefit children with BE by reducing the frequency and severity of acute exacerbations—an independent predictor of a long-term decline in lung function [6]. A randomized controlled trial (RCT) evaluating the effects of an 8-week exercise program in adults with BE reported significant reductions in the number of acute exacerbations over a 12-month period [12]. Moreover, therapeutic exercise programs may also reduce the risk of disabling secondary conditions such as obesity, depression, and anxiety [13].

It is known that children with BE are not sufficiently active to obtain the health benefits associated with regular physical activity (PA). Using an accelerometer to objectively measure daily physical activity, Joschtel and colleagues [14] found children with BE to have low levels of moderate-to-vigorous intensity physical activity (MVPA). Expressed as a percentage of the waking hours, children with BE were sedentary for 57.5% of the time, in light-intensity PA 35.8% of the time, and in MVPA just 6.7% of the time. Indeed, only two children (5.6%) achieved the recommended 60 min of daily MVPA described in the Australian 24-Hour Movement Guidelines for Children and Young People. In contrast, 42% of healthy children in the normative comparison group achieved the guideline. On average, children with BE accumulated 8229 steps/day, well below the recommended 12,000 steps/day. In comparison, daily step counts in healthy children ranged from 11,500 to 14,500 steps/day.

Fundamental movement skill (FMS) proficiency is an important determinant of children’s current and future physical activity status and a significant contributor to individual health and well-being [15]. The development of FMSs early in life is critical to establishing the more complex movement patterns required for participation in all types of play, games, physical activities, and sports [15–17]. Children who are proficient in FMSs are more likely to participate in and enjoy physical activity, achieve higher levels of aerobic fitness, exhibit higher levels of perceived competence and self-esteem, and are less likely to be overweight or obese [15–17]. It has been shown that children with BE exhibit significant delays in their FMS development, thus compromising their ability, confidence, and motivation to participate in PA [18]. In a study of 46 children with BE, only nine (19.6%) achieved their age equivalency for locomotor skills, while just four (8.7%) achieved their age equivalency for object control skills. Fewer than 5% of children demonstrated mastery in the run, gallop, hop, and leap, while fewer than 10% demonstrated mastery for the two-handed strike, overarm throw, and underarm throw. Importantly, children achieving their age equivalency for locomotor or object control skills exhibited higher levels of MVPA, perceived competence, and HR-QoL than children with developmental delays in FMSs.

To address the concerns that children with BE are insufficiently active for health benefits and that developmental delays in fundamental movement skill proficiency are a root cause, we have developed a novel, play-based therapeutic exercise program specifically designed to improve FMS proficiency and aerobic fitness in children with BE. In a pilot RCT involving 21 children with BE, the program significantly improved FMS proficiency, with a 21% increase in locomotion skills and a 31% increase in object control. The effect sizes associated with these improvements were, by convention, large (Cohen’s *d* ≥ 1.2). The program also had a moderate positive effect on aerobic fitness (Cohen’s *d* = 0.5) [14, 19]. We now propose to expand this program to a large multi-center RCT to evaluate the efficacy of our exercise program in reducing the frequency of acute exacerbations of BE. Exacerbation frequency is the only known risk factor for lung function decline in children with BE [20]. Reducing the frequency of exacerbations during childhood through therapeutic exercise may be an important clinical management strategy for preventing a future decline in lung function and respiratory morbidity later in life.

### Objectives {7}

This multi-center RCT is designed to answer the primary research question: does therapeutic exercise decrease the frequency of exacerbations in children with BE? Therefore, the primary objective is to test the efficacy of a novel 8-week, play-based therapeutic exercise program to reduce the incidence of acute exacerbations over 12 months in children with BE aged ≥ 4 and < 13 years. The primary hypothesis is that the proportion of children with no exacerbations at 12 months will be significantly higher in participants receiving an exercise program than in participants in the wait-list control condition.

The secondary objectives are to (1) conduct an economic evaluation of the program and (2) evaluate the effects of the program on FMS proficiency, objectively measured moderate-to-vigorous physical activity (MVPA), cardiorespiratory function (CRF), perceived movement competence, health-related quality of life (HR-QoL), and lung function (FEV1). Our secondary hypothesis is that the program will be cost-effective and efficacious in improving FMS proficiency, daily MVPA, CRF, perceived movement competence, HR-QoL, and lung function.

### Trial design {8}

The BREATH trial is designed as a randomized, controlled, multi-center, observer-blinded, parallel-group (1:1 allocation), superiority trial. Following informed consent/assent, enrolled children will complete baseline assessments (T1). Participants will then be randomized to either the therapeutic exercise program or wait-list control condition. The number of exacerbations and secondary outcomes will be assessed immediately post-intervention at 9 weeks (T2), after 6-months of follow-up (T3), and after 12 months of follow-up (T4).

## Methods: participants, interventions, and outcomes

### Study setting {9}

The RCT will be conducted at the Queensland Children’s Hospital (QCH), Gold Cold University Hospital (GCUH), and Royal Darwin Hospital (RDH) in Australia. QCH is a metropolitan tertiary level hospital providing specialized services to children living in Queensland and northern New South Wales. The QCH has a dedicated Respiratory and Sleep Medicine Department with approximately 15 senior medical officers. GCUH is a tertiary-level facility that includes a pediatric respiratory service with two pediatric respiratory physicians. RDH is a tertiary level hospital and the largest in the Northern Territory that includes a pediatric respiratory service with a respiratory consultant.

### Eligibility criteria {10}

To be eligible for enrollment, participants must be ≥ 4 and < 13 years of age, have a confirmed diagnosis of non-CF BE (by high-resolution chest tomography (HRCT)), or if > 5 years since the last scan, under the regular care of a pediatric pulmonologist; experienced at least one acute pulmonary exacerbation in the past twelve months; and be medically able to participate in the exercise, have their parent provide informed consent, and not be planning to leave the study catchment area in the 12 months following enrollment. Participants will be excluded if they are unstable medically (as advised by the treating pulmonologist), have insufficient cooperation and/or cognitive understanding to perform tasks, have a recent musculoskeletal injury (e.g., muscle strain, sprains, fractures), have an underlying chronic illness other than BE (e.g., CF, neurological or cardiac disorders), are participating in a clinical trial of another investigational drug/devise or interventional therapy, and are unable to attend sessions or return for follow-up assessments.

### Who will take informed consent? {26a}

Participants will be recruited from the respiratory clinics at each study site (QCH, GCUH, RDH). Before or after their appointment, families will be approached by a member of the research team to explain the study and its requirements, gauge interest, and screen for initial eligibility. Parents or carers may request a follow-up at a more convenient time or if they would like additional time to consider participation in the study.

### Additional consent provisions for collection and use of participant data and biological specimens {26b}

On the consent form, parents/guardians will provide consent for researchers to publish or present the data from this trial in a deidentified form. Additionally, if the child is withdrawn, the parent/guardian provides consent to use their data. This trial does not involve collecting biological specimens for storage.

### Interventions

#### Explanation for the choice of comparators {6b}

The therapeutic exercise group will be compared to a wait-list control group receiving usual care, which in the target patient group is currently limited to little or no exercise therapy. Its selection as a comparator is therefore justified. Children allocated to the wait-list control group will receive the exercise program at the conclusion of their 12-month follow-up period.

#### Intervention description {11a}

Children allocated to the intervention will receive the therapeutic exercise program delivered by an accredited exercise physiologist or physiotherapist. The program will include eight 60-min weekly sessions supplemented by a home-based program (two sessions per week for 8 weeks, approximately 20 min per session). This program was designed from the feasibility pilot trial [19]. Group sessions of up to six children will be conducted in community venues at different times and locations to accommodate family schedules. During group sessions, the children will participate in a warm-up and a circuit of six independent games targeting fundamental movement skills and cardiovascular fitness followed by a group game then cool down. The games alternate from higher to lower intensity and can be modified to match each child’s abilities. Children monitor their progress in a “Games Passport” and collect stickers to track their completed sessions. The home program is based on games from the group sessions. Parents receive a detailed manual describing the activities to complete in the home sessions and strategies to support and encourage children to complete the activities. Each week, the child will choose two new activities to complete with their parent at home. Parents document the number of home sessions completed each week on a chart along with the child’s perceived effort [21]. The chart is reviewed by the instructor at the next weekly session.

Prior to leading group sessions, instructors will complete 12-h of comprehensive training led by the exercise physiology and physiotherapy study investigators. Each instructor will review the intervention manual and standard operating procedures and watch videos demonstrating the setup, instructions, modifications, and basic movements for each component of the session. The training will start with the observation of the games with a focus on feedback and reflection. It will progress to leading full sessions with children, including the rehearsal of scripts developed for the home program and tracking. For quality assurance and program fidelity, heart rate data from a sensor will be collected from a sample of sessions, and instructors will complete an essential element checklist after each session. The checklist will include comments about the games completed in the session and confirmation that home session data were collected. Once the intervention has commenced, the lead exercise physiologist or physiotherapist will intermittently visit each community venue to ensure the intervention is being delivered as outlined in the study materials and training sessions.

#### Criteria for discontinuing or modifying allocated interventions {11b}

There will be no special criteria for discontinuing or modifying allocated interventions.

#### Strategies to improve adherence to interventions {11c}

Adherence to the intervention will be monitored via data entered into Research Electronic Data Capture (REDCap) at the completion of each exercise session. To support adherence to the intervention, parents receive a manual that outlines the goals of the BREATH trial and includes home activity sheets. Children are given a home program tracker chart, with stickers, to display in a visible location. Similarly, children add stickers to their Games Passport at the end of each session. Instructors contact parents via text or phone call as a reminder prior to each session.

#### Relevant concomitant care permitted or prohibited during the trial {11d}

Usual concomitant care is permitted for both intervention and wait-list groups. Participation in additional research trials is prohibited during the trial.

#### Provisions of post-trial care {30}

There is no anticipated harm and compensation for trial participation.

### Outcomes {12}

Baseline data collection from parents will include demographics (date of birth, sex, parental age, parental education, household income, and family structure) and relevant medical history. Anthropometric characteristics (height, weight, body mass index (BMI), BMI *z*-score) and medical information (duration and type of cough and current medications) will be measured at each time point. Spirometry data will be extracted using the most recent data in the child’s electronic medical record.

#### Primary outcome

Pulmonary exacerbation will be defined as treatment with antibiotics for any of the following: increased wet cough, dyspnea, increased sputum volume or color intensity, new chest examination or radiographic findings, deterioration in FEV1 percentage by more than 10%, or hemoptysis [22]. Wet cough must be present. Monthly, parental contact and medical review at each assessment time point will document acute respiratory exacerbations.

#### Secondary outcome measures

##### Aerobic fitness

Aerobic fitness will be assessed with a modified shuttle test (MST). The participants move back and forth over a 10-m course at an increasingly faster pace as guided by an audible tone. The test stops when the child is unable to reach the marker by the tone. The assessor will ask the child to rate their perceived exertion using the OMNI scale. The total distance, number of laps, and OMNI score will be recorded [23, 24]. The MST has been validated in children with chronic respiratory conditions [23–25].

##### Fundamental movement skill proficiency

The Test of Gross Motor Development 2nd Edition (TGMD-2) will be used to measure movement competency relative to 12 FMSs subdivided into two subscales: locomotor and object control (ball) skills [26]. The TGMD-2 is a valid and reliable measure of FMS proficiency in children. The TGMD-2 is widely used internationally in typically developing children [27, 28] and children with chronic health conditions [29–31]. Test-retest reliability intraclass correlation coefficients (ICCs) are high for the locomotor and ball skills (ICC = 0.92–0.96) and total TGMD-2 (ICC = 0.77–0.98) score [32].

##### Habitual physical activity

Habitual physical activity will be assessed with accelerometers (ActiGraph GT3X+, ActiGraph Corporation, Pensacola, FL, USA), worn on the child’s non-dominant wrist for 7 days [33, 34]. Accelerometer data will be analyzed using custom software for the determination of daily time spent in sedentary, light, moderate, vigorous, and moderate-to-vigorous physical activity (MVPA). The software will implement previously validated machine learning physical activity classification algorithms developed by Trost et al. [35–37].

##### Perceived movement competence

The Pictorial Scale of Perceived Movement Skill Competence (PMSC) is a valid and reliable tool that assesses the fundamental movement skill competence perceptions of young children [38–40]. The pictorial instrument depicts 12 skills, takes a short time to administer, and has appropriate ICCs (0.76–0.84) [39].

##### Health-related quality of life

Parents will complete the Core Scales of the Pediatric Quality of Life Inventory (PedsQL™) and the Chronic Cough QoL (CCQoL) questionnaires [41, 42]. Children will respond to the child-reported PedsQL™ and the child-reported cough-specific QoL (if aged > 7 years). The PedsQL™ is an efficient multi-dimensional tool that has 23 items. The items address the domains of physical functioning, emotional functioning, social functioning, and school functioning. The PedsQL™ has been used in multiple pediatric BE populations with a high internal consistency (ICC = 0.88 child, 0.90 parent report) [18, 41]. The CCQoL short form has eight questions and is a reliable, valid instrument to assess chronic cough in children [42].

##### Lung function—spirometry

Lung function outcomes will include forced vital capacity (FVC), forced expiratory volume in 1 s (FEV1), FEV1 as a percentage of predicted valued, and the ratio of FEV1/FVC. Spirometry will be completed at routine respiratory appointments at each hospital and extracted from the medical records.

##### Economic evaluation

The within-trial cost-effectiveness analysis and cost-utility analysis will be completed by a health economist to determine whether therapeutic exercise represents “value for money” compared to usual care over a 12-month period from a healthcare provider perspective. Intervention costs will be determined from the project financial records, including costs associated with therapist training and staff time to deliver programs. Pulmonary exacerbation costs (antibiotic treatment and time of clinical staff) will be taken from the Australian Pharmaceutical Benefits Scheme and the appropriate clinical award wage rate. The outcome of interest will be the proportion of children with no exacerbations over 12 months for the cost-effectiveness analysis, and HR-QoL (measured using PedsQL™) for the cost-utility analysis.

#### Participant timeline {13}

**Fig. 1:**
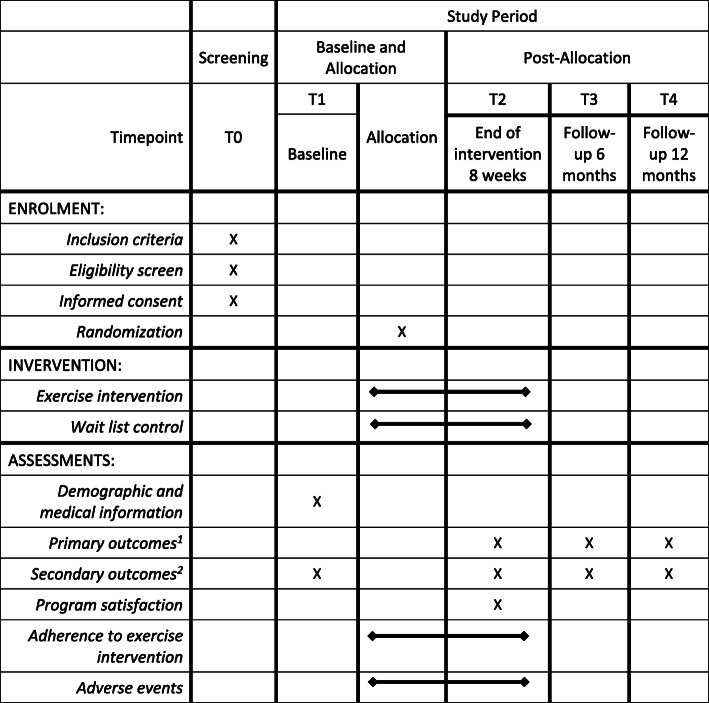
SPIRIT overview of enrollment, interventions, and assessments. ^1^Primary outcome: proportion of children with no exacerbations. ^2^Secondary outcomes: FMS proficiency, objectively measured moderate-to-vigorous physical activity (MVPA), cardiorespiratory fitness (CRF), perceived movement competence, health-related quality of life (HR-QoL), and lung function (FEV1)

Figure 1 displays the participant timeline.

#### Sample size {14}

For the primary objective, the intervention effect is the difference in the percentage of children exacerbation free at 12-months follow-up. Based on the results of an RCT in which the percentage of children exacerbation free in the control group at 12 months was 18% [22], the percentage without an exacerbation over 12-months was estimated to be 20%. Based on the results of an exercise training RCT conducted on 55 adults with BE [12], we expect the therapeutic exercise program to double the percentage of children exacerbation free at 12 months follow-up from 20 to 40%. Assuming a 2-tailed alpha level of 0.05, a sample size of 79 per group (*N* = 158) will provide 80% power to detect the projected 20% group difference in the percentage of children exacerbation free at 12 months follow-up. To account for 10% attrition, a total of 174 participants will be recruited.

#### Recruitment {15}

Participants will be recruited from the respiratory clinics at each study site (QCH, GCUH, RDH). On regularly scheduled clinic days, the clinic manager or research nurse will provide the study coordinator an appointment list of patients with a diagnosis of bronchiectasis ≥ 4 and < 13 years of age. To assess the initial eligibility, families will be asked a series of questions addressing the inclusion and exclusion criteria. Eligible and interested families will be given a participant information sheet and consent form. Following eligibility screening, the attending physician will confirm patient eligibility and provide medical clearance for exercise. To support recruitment, the exercise program will be delivered in local community halls with free parking. The exercise program will also be open to siblings and friends.

### Assignment of interventions: allocation

#### Sequence generation {16a}

Participants will be randomly assigned to either the exercise group or the wait-list control group with a 1:1 allocation using a computer-generated randomization schedule, stratified by site using random permuted blocks sizes 2, 4, or 6.

#### Allocation concealment mechanism {16b}

Participants will be centrally randomized using the randomization module in REDCap [43]. A member of the research team (Research Support Officer) not involved in the delivery of the exercise intervention or the outcome assessments will be given sole privileges to the REDCap randomization module and dashboard. Allocation concealment will be ensured as the other members of the research team, including the chief investigators, will not be given access to the randomization module or the treatment allocation codes.

#### Implementation {16c}

Prior to the commencement of the trial, the randomization schedule will be generated by an independent statistician. Participants will be enrolled in the study by the research staff responsible for recruitment in clinics. Following enrollment, the Research Support Officer will create a REDCap record for each enrolled participant. Following completion of the baseline assessment, the Research Support Officer will assign participants to groups using the REDCap randomization module. Once randomized, the research staff will notify the families of group allocation.

#### Who will be blinded after assignment to interventions {17a}

Trained assessors blinded to the group allocation will collect and process the primary and secondary outcome data at each study site. Data analysts will also be blinded to the group allocation. Within an exercise intervention, it is not possible to blind either therapist or participant; however, the project staff delivering the therapeutic exercise program will not take part in outcome assessments.

#### Procedure for unblinding if needed {17b}

The design is open-label with only outcome assessors and data analysts being blinded, so unblinding will not occur. Any request to unblind a participant must be directed to the Research Support Officer and chief investigator who will liaise relevant ethics committee if required.

### Data collection and management

#### Plans for assessment and collection of outcomes {18a}

Trained assessors blinded to group allocation will collect the data at each study site at each time point. All assessors will be required to meet predetermined inter- and intra-observer reliability standards for each outcome. For the measurement of physical activity (MVPA), accelerometer data will be downloaded and processed into physical activity metrics offline and subsequently imported into the REDCap database.

#### Plans to promote participant retention and complete follow up {18b}

To promote participant retention, participating families will receive a quarterly newsletter with general information about healthy lifestyle behaviors. In addition, for the duration of study participation participants will receive a birthday card from the BREATH Trial team.

#### Data management {19}

All data will be recorded using standardized data entry forms created in REDCap with paper case report forms (CRFs) if needed. All hard copies of participant data will be stored in locked cabinets in secure facilities at the Centre for Children’s Health Research and Menzies School of Health Research. Electronic data will be stored in password-protected databases on Queensland University of Technology’s Research Data Storage Service (RDSS). Research data is encrypted during upload and download and is backed up and duplicated in two geographically dispersed data centers. Only study staff directly involved in the research will have access to the hard copy or electronic files.

#### Confidentiality {27}

All information on study participants will be retained in password-protected files and locked cabinets at the Centre for Children’s Health Research and Menzies School of Health Research. To ensure confidentiality, participants will be allocated an individual trial identification number. Access to this information will only be provided to immediate study staff, unless required by legislative or regulatory agencies and the HREC. Identifiable information will need to be collected to follow up children and their families. Parents/guardians and children will be informed at enrollment that identifiable information is being collected but will not be disclosed to anyone outside the study team. The identifiable information will specifically be name, address, and date of birth. This information will be removed from the dataset at the end of the study, and any future use of the data will be in de-identified form. All data presented in any publicly accessible notes and/or publications arising from the research will be presented in aggregate form only. No individual information or identifying information will be presented. Field notes will only reference the participants’ study identification number. In the state of Queensland, the law requires that all research data collected on children be stored indefinitely and cannot be destroyed.

#### Plans for collection, laboratory evaluation, and storage of biological specimens for genetic or molecular analysis in the current trial and for future use {33}

This trial does not involve collecting biological specimens for storage.

## Statistical methods

### Statistical methods for primary and secondary outcomes {20a}

The statistical analyses will follow standard principles for RCTs using two group comparisons including all participants on an intention-to-treat basis. For the primary objective, a binary regression model with a suitably chosen link function (e.g., logistic) will be used where the target variable is whether or not an exacerbation occurred over the 12-month study period for each subject. The main covariate of interest is the treatment group (exercise vs. wait-list control). Other covariates potentially influencing the target variable such as age and sex will be considered. Therefore, the statistically determined treatment effect will be appropriately adjusted for unbalanced data with respect to the other important covariates retained in the regression model. Between-group differences in secondary outcomes—aerobic fitness, habitual PA, perceived competence, HR-QoL (PedsQL), and lung function (FEV1)—will be tested immediately post-intervention, at 6 months follow-up, and at 12 months follow-up using linear regression with treatment group (exercise vs. wait-list control) as the main effect and baseline measures of the respective outcome, along with other potential confounders (e.g., age, sex), as covariates.

### Interim analyses {21b}

Interim analyses are not planned.

### Methods for additional analyses {20b}

No other additional analyses are planned.

### Methods in analysis to handle protocol non-adherence and any statistical methods to handle missing data {20c}

Data will be analyzed using an intention-to-treat approach. Significant protocol non-adherence and drop-out are not anticipated, and any expected drop-outs are factored into the target sample size. Missing data will be imputed using multiple imputation procedures under the assumptions of missing at random (MAR) and multivariate normality. The imputation step will be implemented using the Markov chain Monte Carlo (MCMC) method in SAS PROC MI. PROC MIANALYZE will be used to combine the results into a single set of parameter estimates, standard errors, and test statistics [44].

### Plans to give access to the full protocol, participant-level data, and statistical code {31c}

The dataset, protocol, and statistical code (if applicable) will be made available to appropriately qualified investigators upon reasonable request to the principal investigator. The research team will have exclusive use of the data for a period of 12 months from the end of the project or until the data is published.

### Oversight and monitoring

#### Composition of the coordinating center and trial steering committee {5d}

Research staff from the Children’s Physical Activity Research Group (CPARG) at the Queensland Centre for Children’s Health Research in Brisbane will provide study coordination services and be responsible for the day-to-day running of the trial. The Research Support Officer from CPARG will review the eligibility, enrollment, and consent for each participant; independently verify data entered into REDCap; assign the participants to groups using the REDCap randomization module; monitor the assessment time points for scheduling; and review the data for completeness. The Research Project Officer from CPARG will independently review the data from the study assessments and exercise sessions, maintain the assessment equipment, support the research assistants and instructors, and oversee the exercise site organization. A trial steering committee comprising the chief investigators and associate investigators from each site will meet monthly via a videoconference in the first year, and quarterly in study years two through four of the trial, to ensure protocols are properly established and implemented and that project milestones are completed on time.

#### Composition of the data monitoring committee, its role, and reporting structure {21a}

A data monitoring committee was deemed unnecessary as the study is relatively short in duration and does not involve significant safety concerns, risks to participants, or complexity.

#### Adverse event reporting and harms {22}

Surveillance for adverse will occur for the duration of the exercise program. Any minor adverse event (e.g., as minor injury) associated with participation in the therapeutic exercise program will be screened on a weekly basis by the exercise therapists by verbal questioning and recorded on the session checklist. Risk assessments with mitigation strategies will be completed prior to implementing the therapeutic exercise program. It will be the responsibility of the chief investigator to ensure that all adverse events, including serious adverse events (SAEs) events, are documented and accurately reported. All the study staff will be trained in the requirements and procedures of SAE and medically significant event documentation and reporting.

Serious adverse events (SAEs) are not anticipated given the low-risk exercise intervention but are defined as an experience that results in death, is life-threatening, requires unexpected in-patient hospitalization or prolongation of existing hospitalization, or results in persistent or significant disability/incapacity. Potential adverse events (AEs) associated with physical activity may include a fall, minor injury, exacerbation of existing symptoms, muscle soreness, or impact from sporting equipment. A protocol-related adverse event is an AE occurring during the study that is not related to the intervention but is considered by the investigator or medical monitor to be related to the research conditions, i.e., related to the fact that a subject is participating in the study. This may include AEs due to study procedures such as clinical diagnostic procedures.

Any SAE must be reported to the chief investigator within 24 h of becoming aware of the event. The project manager will complete the SAE form with as much information regarding the event that is available to them at the time. Telephone reports will be followed by a full report including copies of relevant medical records and other documents. SAEs are to be reported to the HREC within 24 h of notification. Follow-up reports must also be forwarded to the HREC as specified by the relevant HRECs in each jurisdiction. Participants and/or the infant’s primary carer will be asked to notify the study coordinators of any serious adverse events (SAEs) that may occur during the active study period.

#### Frequency and plans for auditing trial conduct {23}

A Project Management Group comprising the chief investigators and associate investigators at each site will meet monthly to review the trial conduct. These meetings will include the research staff from all sites. For quality assurance purposes and program fidelity, exercise therapists will complete a brief checklist after each session for the purposes of evaluating adherence to the program “essential elements” and to note any issues or difficulties encountered during the session. Additionally, the senior exercise therapist will complete on-site observations of program delivery on a quarterly basis. To monitor fidelity to the home-based program, parents/carers will complete a weekly training log provided at the first session.

#### Plans for communicating important protocol amendments to relevant parties (e.g., trial participants, ethical committees) {25}

Protocol amendments will be submitted to the lead ethics committee for review. The amended protocol will be distributed to sites by the Research Support Officer and updated on the clinical trial registry (ANZCTR).

#### Dissemination plans {31a}

The results of the BREATH trial will be disseminated through peer-reviewed journal publications, conference presentations, and via the ANZCTR.

## Discussion

Pediatric BE is a significant contributor to global mortality, morbidity, and healthcare utilization, yet there is a paucity of research on non-pharmaceutical interventions that aim to improve HR-QoL and impact disease progression for this population [1–3, 6]. It has been established that children with BE have delayed FMS proficiency leading to insufficient physical activity for health benefits [18]. A pilot RCT involving 21 children with BE demonstrated that a therapeutic exercise program is feasible and potentially efficacious in improving FMS proficiency and fitness [19]. A larger, fully powered RCT is now needed to determine the long-term impacts of the program, specifically the effects on acute exacerbations and HR-QoL.

This multi-center RCT will establish and test a highly scalable, therapeutic exercise program to reduce the proportion of children with no exacerbations of BE over 12 months and improve movement competency, HR-QoL, and exercise capacity. Improvements in these outcomes are likely to have an immediate positive impact on physical functioning, quality of life, and future health. BE exacerbation is the only known risk factor for lung function decline in this patient group. Thus, reducing the frequency of exacerbations during childhood through therapeutic exercise may be an important clinical management strategy for preventing a future decline in lung function and respiratory morbidity later in life.

This RCT is significant because it is the first fully powered RCT to test the effects of a therapeutic exercise program to prevent exacerbations and improve FMS proficiency, fitness, and HR-QoL in children with BE. By implementing a developmentally appropriate, play-based exercise program tailored to the individual needs of children with BE, the results have the potential for a major paradigm shift in the way in which therapeutic exercise is prescribed and implemented in children with chronic respiratory conditions. The exercise program can be readily translated. It does not require expensive equipment and can be delivered in a variety of settings, including the participant’s home. The program of research has strong potential for translation to other pediatric patient groups with similar needs for exercise therapy, including those with obesity, childhood cancers, and neurological conditions such as cerebral palsy. While exercise is well recognized to be universally beneficial, this RCT is required because it will provide long overdue level 1 evidence regarding the efficacy and cost-effectiveness of tailored therapeutic exercise programs for children with BE.

## Trial status

The recruitment of participants commenced at all study sites on the 17 February 2021 and is planned to continue until 30 June 2022 (protocol version 4, 10 August 2021).

### Authors’ contributions {31b}

ST conceived the study, its design, and coordination. KO’G, IM, VG, GMc, and ABC supported the design and submission to the National Health and Medical Research Council. CD oversaw the analysis plan. TL oversaw the economic analysis plan. DSKB, EB, and TJ participated in the development of the intervention. ST, CT, and DSKB developed the measurement protocols and REDCap research database. TJ, CT, and DSKB will assist with the recruitment, assessment, and intervention. All authors read and approved the final manuscript.

## Abbreviations

AE: Adverse event
ANZCTR: Australian and New Zealand Clinical Trials Register
BE: Bronchiectasis
BMI: Body mass index
BREATH: Bronchiectasis - Exercise as Therapy
CCHR: Centre for Children’s Health Research
CCQoL: Chronic cough QoL
CF: Cystic fibrosis
CPARG: Children’s Physical Activity Research Group
CRS: Case report from
CSLD: Chronic suppurative lung disease
FEV1: Forced expiratory volume in 1 s
FMS: Fundamental movement skill
FVC: Forced vital capacity
GCUH: Gold Cold University Hospital
HRCT: High-resolution chest tomography
HR-QoL: Health-related quality of life
ICC: Intraclass correlation coefficient
MAR: Missing at random
MCMC: Markov chain Monte Carlo
MST: Modified shuttle test
MVPA: Moderate-to-vigorous intensity physical activity
PA: Physical activity
PedsQL™: Core Scales of the Pediatric Quality of Life Inventory
PMSC: Pictorial Scale of Perceived Movement Skill Competence
QCH: Queensland Children’s Hospital
QOL: Quality of life
RDSS: Research Data Storage Service
REDCap: Research Electronic Data Capture
RCT: Randomized controlled trial
RDH: Royal Darwin Hospital
SAE: Serious adverse events
TGMD-2: Test of Gross Motor Development 2nd Edition
